# Role of Rhinovirus C in Apparently Life-Threatening Events in Infants, Spain

**DOI:** 10.3201/eid1509.090453

**Published:** 2009-09

**Authors:** Cristina Calvo, M. Luz García, Francisco Pozo, Noelia Reyes, Pilar Pérez-Breña, Inmaculada Casas

**Affiliations:** Hospital Severo Ochoa, Leganés, Madrid, Spain (C. Calvo, M.L. García); National Center for Microbiology, Institute of Health Carlos III, Madrid (F. Pozo, N. Reyes, P. Pérez-Breña, I. Casas).

**Keywords:** Picornavirus, rhinovirus, HRV-C, infants, apparent life-threatening events, viruses, dispatch

## Abstract

To assess whether infants hospitalized after an apparently life-threatening event had an associated respiratory virus infection, we analyzed nasopharyngeal aspirates from 16 patients. Nine of 11 infants with positive virus results were infected by rhinoviruses. We detected the new genogroup of rhinovirus C in 6 aspirates.

Human rhinovirus (HRV) is 1 of the most common agents associated with upper and lower respiratory tract infections in children and infants (*1*) and is a major trigger of asthma exacerbations (*2*). Recently, molecular methods have shown substantial phenotypic variation of HRV and identified a novel HRV genogroup provisionally named HRV-C (*3*). Severe asthma exacerbations in children have been associated with this new genogroup of rhinoviruses. Genogroup C could be resistant to a new candidate group of antipicornavirus drugs, including pleconaril (*4*).

Apparently life-threatening events (ALTEs) in infants are associated with bronchiolitis or infections in up to 6% of patients by diagnosis after hospital admission (*5*). We assessed the relation between ALTEs and respiratory virus infection in a secondary hospital in Spain.

## The Study

Our study was part of a systematic prospective study to assess the epidemiology of respiratory virus infections in children admitted to the Severo Ochoa Hospital (Leganés, Madrid Province, Spain).We conducted a specific study to determine the incidence of respiratory virus infections in all infants admitted after ALTEs during November 2004–December 2008. An ALTE in a child <1 year of age was defined as an episode that is frightening to the observer and characterized by some combination of apnea, color change, marked change in muscle tone, choking, or gagging so the observer fears the infant has died (*6*).

Nasopharyngeal aspirate (NPA) specimens were acquired from each eligible patient at the time of hospital admission (on Monday–Friday). Samples were sent for virologic study to the Influenza and Respiratory Virus Laboratory (National Centre for Microbiology, Institute of Health Carlos III, Spain). Specimens were processed within 24 hours after collection.

Total nucleic acids were extracted from 200-µL aliquots by using a QIAamp MinElute Virus Spin Kit in a QIAcube automated extractor (QIAGEN, Valencia, CA, USA). Simple or multiplex reverse transcription–nested PCR assays (RT-PCR) previously described (*7**–**9*) were used to assess the virus diagnosis, including 16 respiratory viruses or groups of viruses. Degenerated primers for HRV and enteroviruses were designed between the 3′ end of the 5′ noncoding region (NCR) and the viral protein (VP) 4/VP2 polyprotein gene (TCIGGIARYTTCCASYACCAICC-3′ and CTGTGTTGAWACYTGAGCICCCA-3′). HRVs from positive samples were identified by sequencing and phylogenetic analysis of these sequences. Amplified products (about 500 bp, depending on HRV serotype) were purified and sequenced in both directions by using an automated ABI PRISM 377 model sequencer. Partial sequences of HRV have been submitted to GenBank (accession nos. FJ841954–FJ841957, FJ841959–FJ841961, EU697826, and EU697832). Appropriate precautions were implemented to avoid false-positive results by carryover contamination. Positive results were confirmed by testing a second aliquot of the sample stored at –70ºC.

Sixteen infants (8 of each sex) were enrolled in the study. All patients were <5 months of age (range 7 days–5 months, mean age 7.6 weeks, median 4 weeks). Twelve infants had rhinorrea, cough, and distress signs (Table). A total of 11 (69%) NPA specimens were positive for at least 1 viral agent. For 9 of these patients, positive results for HRV were confirmed, and for the other 2 patients, respiratory syncytial virus was detected.

**Table:** Characteristics of infants with ALTEs, Spain, November 2004–December 2008*

Laboratory no.	Sex/age, wk	Admission date	Clinical signs	Discharge diagnosis	Virus	Prematurity
SO3970	M/4	2004 Nov	Cough, rhinorrea, loss of consciousness, flaccidity	Choking	HRV-B	No
SO4891	M/4	2005 Oct	Cyanosis, loss of consciousness, flaccidity	Cyanosis	No	Yes (35 wk)
SO4923	M/15	2005 Nov	Apnea, flushing	Cyanosis + URTI	HRV-A	Yes (36 wk)
SO4998	F/8	2005 Nov	Choking, flushing, distress	URTI + GERD	HRV-B	No
SO5260	F/9	2006 Apr	Cough, choking	GERD	No	No
SO5355	M/6	2006 Jun	Apnea, cyanosis	URTI + GERD	No	No
SO5529	F/4	2006 Nov	Apnea, congestion, cyanosis	Bronchiolitis + GERD	RSV	No
SO5749	M/24	2007 Mar	Choking, cyanosis	Wheezing	No	No
SO5797	F/6	2007 Mar	Apnea, flushing	Choking + GERD	HRV-C	No
SO5854	F/6	2007 Apr	Cough, rhinorrea, apnea	URTI + GERD	HRV-C	No
SO5896	M/13	2007 Sep	Apnea, rhinorrea	Bronchiolitis + GERD	HRV-C	No
SO6012	F/7	2007 Oct	Cough, distress, apnea	URTI	RSV-A	No
SO6666	M/4	2008 Oct	Cyanosis, choking	Choking	HRV-C	No
SO6813	F/6	2008 Nov	Apnea, flaccidity	Apnea + GERD	HRV-C	No
SO6819	M/6	2008 Nov	Cough, rhinorrea, flushing	URTI + GERD	HRV-C	No
SO6816	F/1	2009 Jan	Choking, flushing	Choking	No	No

*ALTEs, acute life-threatening events; URTI, upper respiratory tract infection; RSV, respiratory syncytial virus; HRV, human rhinovirus; EV, enterovirus; GERD, gastroesophageal reflux disease.

Phylogenetic analyses of 9 sequences obtained from patients showed distribution of HRV in 3 clusters. Three sequences were included in previously characterized clades, defined by HRV group A (HRV-A, SO4923–EU697826) and B (HRV-B, SO3970–FJ841954 and SO4998–EU697832). Sequence from patient SO4923 had a low sequence similarity with the other serotypes of HRV-A. In contrast, sequences from patients SO3970 and SO4998 were closely related to HRV-35 and HRV-79, respectively. Six sequences were included in the third group corresponding to the new HRV-C: SO5854, SO6666, SO5797, SO6819, SO5986, SO6813- FJ841955-57 and FJ841959-61) (*3**,**10*) (Figure). Different genotypes (collectively called HRV-Cs) were identified in 6 NPA specimens from children with ALTEs (67% of total HRV). Two received cardiopulmonary resuscitation at home; for these 2 patients, a respiratory syncytial virus and an HRV-C were identified. All 16 children survived.

**Figure:**
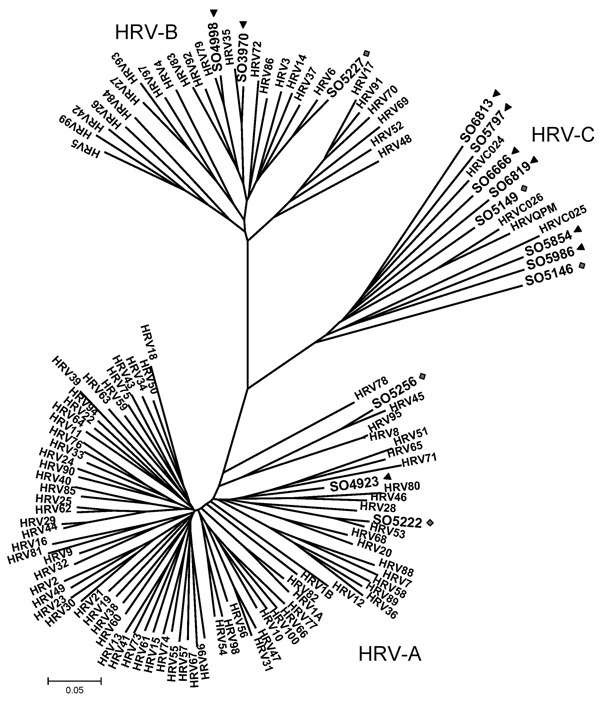
Phylogenetic analysis of 5′ noncoding region and viral protein (VP) 4/2 coding region of 9 human rhinoviruses (HRVs) identified in infants with apparently life-threatening events in Spain, November 2004–December 2008. Phylogeny of nucleotide sequences (≈492 bp) was reconstructed with neighbor-joining analysis by applying a Jukes-Cantor model; scale bar indicates nucleotide substitutions per site. Included for reference are sequences belonging to the novel genotype reported previously (QPM and 024, 025, 026 [*11*]) and all HRV-A and -B serotypes available in GenBank. Significant bootstrapping is indicated.

## Conclusions

The most common discharge diagnoses reported for ALTEs are gastroesophageal reflux disease (GERD), unknown causes, seizures, and lower respiratory tract infections (*11*). Our series suggests that ALTEs of previously unknown etiology could be related to HRV infections. Rhinovirus infections are known to be a major cause of illness and hospital admission for young children, particularly infants <2 years of age (*12*). Detection of viral genomes by nested RT-PCR in NPA specimens led us to analyze the effect of HRV infections in different clinical situations. Respiratory infections associated with HRV might play a major role in young infants, probably with few clinical signs, and might contribute to apnea as a first manifestation. GERD is the most frequent hospital discharge diagnosis in published series (*5**,**11*). For our patients, GERD also was the most frequent clinical diagnosis (9 patients), but for 7 of them, a respiratory virus was identified. We cannot conclude whether GERD is a risk factor for apnea or whether signs are so nonspecific that diagnoses could be confused.

Alternatively, the new HRV-C group could account for as many as a quarter or even half of HRV infections (*4**,**13*). In children, it has been associated with bronchiolitis, wheezing, and asthma exacerbations severe enough to require hospitalization; the percentage of these children with hypoxia was substantial (*13*). In a case–control study, Khetsuriani et al. (*4*) found HRV-C only in case-patients, supporting the pathogenic role of this genogroup. They considered that HRV-C infections could be associated with more severe clinical manifestations than infections with other HRV genogroups A and B. These data could also support the role of HRV-C in infants with ALTEs found in this work.

Although we had no control group for our patients, we recently published a study of a cohort of 316 newborns up to 6 months of age tested weekly for respiratory diseases (mainly upper respiratory tract infections), coincident in age and time with our patients (*14*). HRV was present in 5 (3.6%) of 72 infants tested. Two viruses were genetically identified as HRV-C, demonstrating they form distinct genetic clusters, and no genetic similarity was obtained with the ALTE–related HRV-C viruses. In addition, a second group of asymptomatic children of different ages but in coincident epidemic seasons was studied. The group of children with HRV was substantially smaller than the group of children with respiratory disease (*15*).

Viral infections could play a major role in ALTEs. Rhinoviruses, especially HRV-C, could cause a respiratory infection with few symptoms in young infants and could trigger ALTEs in this age group. Therefore, HRVs and posterior genotyping should be included in studies of the etiology of ALTEs to help identify the true relevance of HRV-C infection to these episodes.
